# Breast cancer: are long-term and intermittent endocrine therapies equally effective?

**DOI:** 10.1007/s00432-020-03264-0

**Published:** 2020-05-29

**Authors:** Jutta Engel, Gabriele Schubert-Fritschle, Rebecca Emeny, Dieter Hölzel

**Affiliations:** 1 Munich Cancer Registry (MCR), Institute for Medical Information Processing, Biometry and Epidemiology (IBE), Ludwig-Maximilians-University (LMU), 81377 Munich, Germany; 2 The Dartmouth Institute for Health Policy and Clinical Practice, Geisel School of Medicine at Dartmouth, Lebanon, NH 03756 USA

**Keywords:** Breast cancer, Metastasis, Long-term endocrine adjuvant therapy, Over-treatment, Survival

## Abstract

**Purpose:**

In breast cancer (BC), the duration of endocrine adjuvant therapies (AT) has been extended continuously up to 10 years. We present an alternative explanation for the effect, which could enable shorter treatments.

**Method:**

The relevant literature on chemoprevention and (neo-)adjuvant therapy was reviewed. Data for initiation and growth of primary and contralateral BCs and their metastases (MET) were considered. Also, population-based data from the Munich Cancer Registry for MET-free survival, time trends of MET patterns, and survival achieved by improved ATs are used to estimate all events in the long-term follow-up.

**Results:**

Extended ATs (EAT) that continue after 1, 2, or 5 years reduce mortality only slightly. The effect is delayed, occurring more than 5 years after extension. EATs does not affect the prognosis of 1stBCs, they preventively eradicate contralateral 2ndBCs and thus their future life-threatening METs. Because chemoprevention can eradicate BCs from the smallest clusters to almost detectable BCs, ATs can be temporarily suspended without imposing harm. Results equal to EATs can be achieved by short-term ATs of the 1stBC and by repeated neo-ATs targeted at the indefinitely developing 2ndBCs. Considering this potential in de-escalation, a 70–80% reduction of overtreatment seems possible.

**Conclusion:**

Knowledge of initiation and growth of tumors with known effects of neo-ATs suggest that intermittent endocrine ATs may achieve the same results as EATs but with improved quality of life and survival because of fewer side effects and better compliance. The challenge for developments of repeated ATs becomes: how short is short enough.

## Introduction

The goal of adjuvant therapies (ATs) is to reduce BC mortality. This is only possible by reducing life-threatening metastases (METs), either by eradication or prevention. ATs have improved the prognosis of BC significantly over the past decades. 15-year tumor-specific survival should now exceed 80% in comparison to 55% decades ago (Hölzel et al. 2017; Noone et al.; Welch et al. 2016). Endocrine ATs with tamoxifen and aromatase inhibitors showed an additional delayed survival benefit of less than 3% when extended to 5, 10 and more years (Bartlett et al. 2019; Davies et al. 2013; Goss et al. 2016; Gray 2013). This broad range of treatments has been considered extended AT (EAT) and guidelines already recommend 10 years of ongoing ATs (Burstein et al. 2019; Cardoso et al. 2019). Analysis of population-based data from the Munich Cancer Registry (Munich Cancer Registry) with long-term follow-up and changing MET patterns (Hölzel et al. 2017) have revealed principles of MET that suggest a critical review of EATs. The aim of this review is to explain the proven benefit of EAT and to discuss functionally equivalent AT strategies of intermittent ATs based on principles of tumor biology and empirical knowledge.

## Methods

The article is based on a review of pertinent randomized controlled trials and meta-analysis of BC mortality to be considered for explaining the effect of EATs. First, the effects of neoadjuvant and ATs are considered. In ATs, we focus on the important distinction between the effects of delayed AT versus EATs. The second major focus concerns the initiation and growth of BCs and their METs. Chemoprevention is particularly relevant, because it provides data for the risk reduction of first and second BCs (1stBC/2ndBC) and for their growth duration.

Third, population-based data of the Munich Cancer Registry (MCR) are taken into account, whose versatile analysis ultimately led to the results presented herein, including the generation of the hypothesis that EAT maybe considered as overtreatment. We consider MET-free survival with a follow-up of 20 years and more, as well as time trends of the MET pattern and survival achieved by improved ATs. Analyses of MCR data have revealed relevant principles of MET and their treatment. Known data on the incidence of 1st/2ndBCs are also considered. These data and combinations of the effects of short-, long-term, delayed and preventive neo-ATs provide an explanation for the remarkable delay in effect of EATs.

## Results

### Growth and initiation of breast cancer

**Fig. 1:**
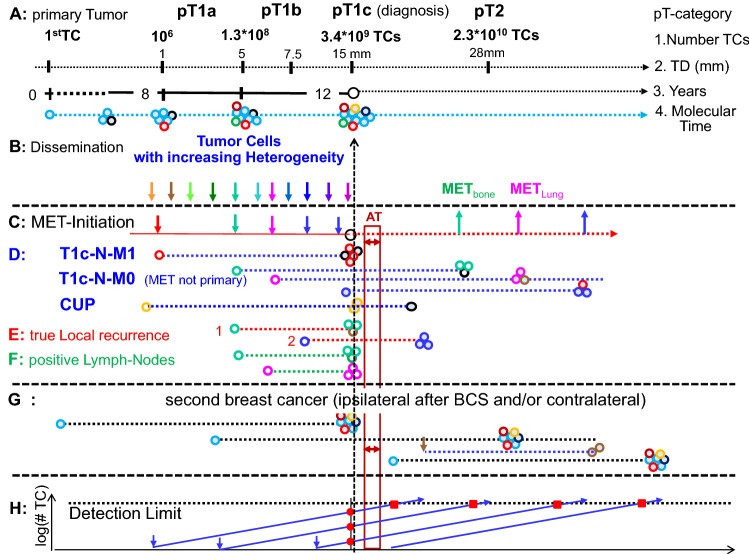
Initiation of metastases. Growing BCs (a) can initiate metastases (c, d), true local recurrences (e), positive lymph nodes (f) over time through dissemination of heterogeneous tumor cells (b). If not diagnosed synchronously, these secondary foci as well as 2ndBC (g) are already prevalent at diagnosis (h) and can be affected by ATs which are short compared to the growth duration of METs. *TC* tumor cell, *TD* tumor diameter, *MET* metastasis, *AT* adjuvant therapy, *BCS* breast conserving surgery

Growing BCs can be described in four ways, by the number of tumor cells (TC), the duration of growth, the diameter of BCs and a molecular timeframe which distinguish phases with relevant mutations that disseminated TCs can inherit (Fig. 1a) (Yates et al. 2017). The growth of BCs has been estimated from mammography screening. For 60–70 year old patients, the median volume doubling (VD) time for tumor growth from 10 to 20 mm is 143 days, with a variation from 65 to 308 days for the 25th and 75th percentile (Weedon-Fekjaer et al. 2008). Applying these percentiles, a pT1a-BC of 3 mm diameter would grow to a pT2-BC of 28 mm (the average of pT2) after 9.6 VDs within 1.7/3.8/8.2 years, respectively. The biennial screening with 20–25% interval cases confirms the variability.

If we assume a constant growth with these three percentiles from the first TC, then a pT1c-BC will be reached after approximately 31.5 VDs or after 5.6/12.3/26.6 years. Population-based estimates reveal only about 7% fast-growing triple negative BCs, that is, even the growth rate of hormone receptor positive (HR +) BCs varies by a factor of 10 and more. These growth rates are plausible, because in prevention trials the incidence reduction persists for at least 15 years after the end of chemoprevention (Cuzick et al. 2015). Therefore, contralateral 2ndBCs are already prevalent and may be diagnosed within the next 12.3 years after the removal of the 1stBC, 2% synchronous BCs are the first of them. 2ndBCs that appear after 12.3 years are increasingly initiated year after year following the removal of the 1stBC (Fig. 1g).

### Initiation and growth of MET

The initiation of METs can be read from survival data. According to our data, the tumor-specific 15-year survival of pT1- and pT2-BCs was at 76% and 53% in the period 1988–1997. Therefore, when a pT1c-tumor with a diameter of 15 mm is reached, at least 24% already had early initiated METs. However, more than 23% additional METs will be initiated while growing up to 28 mm, which totals 47% METs that could not be eradicated by former use of ATs. A Gompertz function can be fitted for the S-shaped relation of tumor diameter and tumor-specific mortality, which results as the complement to 100% in the following: relative 15-year survival (%) = 100 − 58.39 × exp(− 4.46 × exp(− 0.071 × TD)) for tumor diameter (TD) ≤ 90 mm (Engel et al. 2019). With each additional millimeter, a growing BC initiates 0–1.5% additional METs which are diagnosed in a median time span of 28 months (Munich Cancer Registry) before death.

**Fig. 2:**
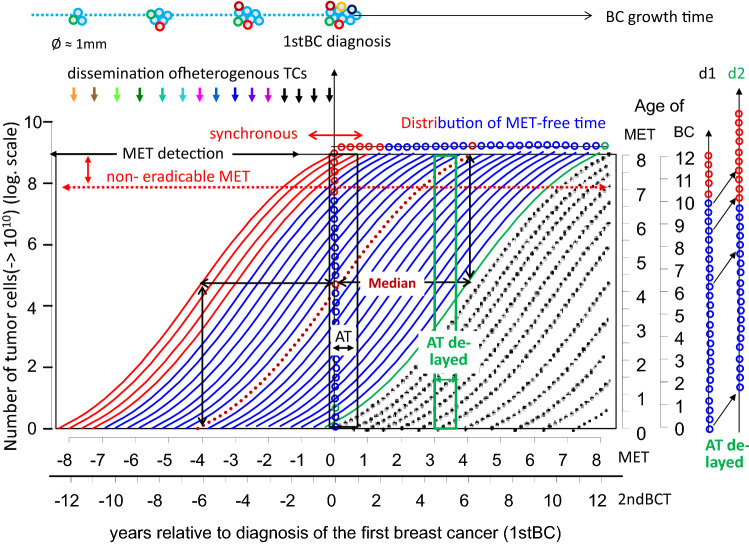
Growth trajectories (GT) for BCs and METs. The time scales (median growth time 8 years for METs and 12 years for 2ndBCs) distinguishes times before and after BC diagnosis. Early initiated, synchronously diagnosed and no longer eradicable METs (red GTs), a median GT (brown dotted) and a late-initiated GT (green) are outlined. The age scale represents the age of prevalent METs at BC diagnosis. In addition, age distributions of METs are outlined in patient cohorts with immediate or delayed onset of ATs. Also outlined is the short duration of today’s ATs (black and green rectangle) compared to the growth period of METs. With the time scale for 2ndBCs, GTs are to be interpreted analogously. The dotted GTs represents 2ndBCs initiated after BC diagnosis (*BC* breast cancer, *MET* metastasis, *AT* adjuvant therapy, *TC* tumor cell)

**Fig. 3:**
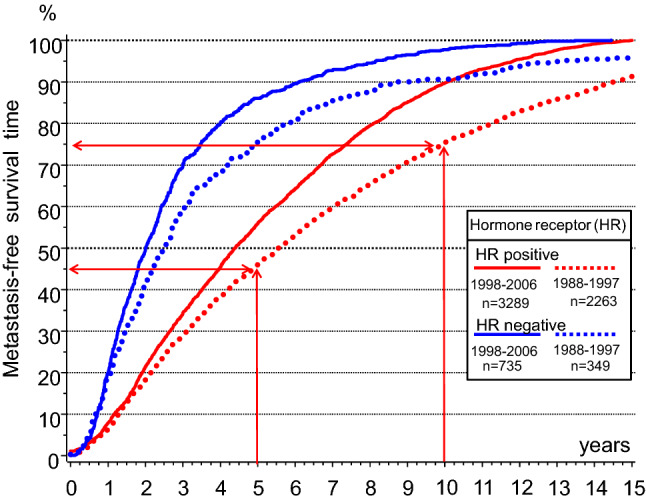
Distribution functions of MET-free intervals. The dependency on hormone receptor status from BCs and on time periods with different follow-up is to be considered (*n* = 6636 of T-N-M0 BCs)

Initiation, growth and treatment effects can be explained by growth trajectories, with a log scale for the number of TCs in relation to time (Fig. 2). Two growth sections need to be distinguished; before and after the diagnosis of a BC. METs of primary advanced BCs have grown after initiation parallel to the BC and are diagnosed simultaneously. In primary M0 findings, the last MET initiations take place shortly before the BC removal. The sum of growth times before and after diagnosis are the same as for a median MET initiation. The distribution function of the MET-free survival time (Fig. 3) results in a median time of about 4 years. Taken together, a MET should grow a median of 8 years before its detection, or 93 days for one VD (Hölzel et al. 2010). This is long compared to the few months of AT regimes which act on MET (Fig. 2).

This MET process is correct as long as there is no initiation of METs after R0 resections (DeMichele et al. 2017). But it is a contradiction to the dormancy hypothesis, according to which METs can be initiated with time delays. But this explanation is not plausible. If circulating or dormant TCs could initiate delayed METs, then the shortening of each AT from years to a few months would have shown disadvantages, but until today, none have been reported. In addition, a long delay in initiated METs would have to be more often fast-growing triple negative tumors (Fig. 3). That is also not true, and therefore, TCs most likely do not cause clinically relevant METs after BC removal.

### Effects of neoadjuvant and adjuvant therapies

Despite successful ATs, the survival after MET has not greatly improved and has now a median of about 28 months. Improving survival means successfully targeting occult METs. METs of HR + BCs grow slower than HR-BCs. About 50/90% of MET of HR + BCs occur within a MET-free period of 5.6/15 years in comparison to 2.5/8.5 years for HR- BCs (Fig. 3). AT trials show no effect in the first few months. A possible reason is the exclusion of advanced BCs by staging, so that the remaining MET need more time up to detection (Fig. 2). Since diagnosed METs remain ineradicable, this also applies to these larger undetected METs which occur in the first few months following BC diagnosis. Only then, after about 6 months or 1–2 VDs of METs do MET-free survival curves open like scissors. The mortality reduction is delayed for another 28 months. Even if treatments lasted only 1 year, there was a reduction in METs after 5 and 10 years (Early Breast Cancer Trialists’ Collaborative Group 1992).

The effectiveness of ATs is also manifested in delayed onset. Even a short delay of AT by 2 months after surgery leads to worse outcomes, because some prevalent METs grow to a no longer eradicable size in that short period of time, which is otherwise prevented by ATs by the annihilation of METs within one VD (Gagliato et al. 2014; Yung et al. 2020). The distribution d1, outlined in Fig. 2 represents a cohort of patients in whom METs were continuously initiated. At BC diagnosis, there are occult METs, from smallest clusters to almost detectable foci. The altered age structure of occult METs when initiations of AT are delayed describes distribution d2 with less tiny and more already detected METs.

Two studies show the effect of delayed starts of tamoxifen treatment after 2–5 years of tumor-free survival (Delozier et al. 2000; Veronesi et al. 2010). In a cohort of patients, when ATs start after a 5 year delay, almost 50% of all patients who are expected to develop METs during the first 5 years are already excluded (Fig. 3). The effect of these delayed ATs on METs which are still occult after 5 years is similar to the scissors-like opening of immediately initiated ATs for all METs. However, there are no longer small TC clusters, because they have grown in size during the time of the treatment delay. This comparability confirms that occult METs of various ages were eradicated very quickly by ATs until they reach a no longer eradicable size.

### Effects of extended adjuvant endocrine therapies

Studies investigating EATs up to 10 years (Bartlett et al. 2019; Davies et al. 2013; Goss et al. 2016; Mamounas et al. 2019) showed no survival effect for 5 years. This implies that EATs have no effect on METs of 1stBCs, although 55% or 25% of METs will occur after 5 or 10 years, respectively (Fig. 3). The ineffectiveness is plausible if there are resistant METs. But there is no tumor biology model so far that explains delayed eradication of METs. That is why the question arises, which effect will be achieved by the extension of ATs from 1 or 2 years to 5 years. 1–2 years of ATs was shown to improve survival even after 10 years (Early Breast Cancer Trialists’ Collaborative Group 1998; Ekholm et al. 2016). Comparisons of 2 versus 5 years of tamoxifen(Swedish Breast Cancer Cooperative Group 1996) and also placebo-controlled comparisons after 2 years of AT (Rutqvist and Johansson 2007) show that survival does not change as was also the case of immediate receipt of AT after 1 year. Additionally, the extension of ATs after 1 or 2 years have a delayed effect as in the 5/10-year comparisons, although 95 or 85% of METs occur later (Fig. 3) (Bartlett et al. 2019; Davies et al. 2013). A delay in MET eradication of more than 5 years is also evident in the 5-year placebo comparison of tamoxifen (Early Breast Cancer Trialists’ Collaborative Group 2011). Therefore, the phase of more than 5 years of ineffectiveness of EATs is very likely to be constant and transferable with EATs used after one year.

### Effects of successful adjuvant therapies over decades

Changes in MET patterns observed in the MCR over decades of successful AT use is an important aspect (Hölzel et al. 2017). In comparison to MET patterns of earlier decades and today’s primary advanced BCs, bone and lung METs have been reduced by about 50% and 30%, respectively. METs are destroyed proportionally from the smallest cluster to a non-eradicable MET size. Alternatively, a clinically relevant reduction of liver or CNS METs is not recognizable. Therefore, the inability of ATs to act equivocally in any micro-environment of MET is one limitation of today’s ATs. Another limitation is that even the smallest clusters show acquired resistance, because even very late bone or lung MET occur which would have to have been very small during AT. Of particular note is that the historical success of ATs is almost independent of BC size. It is achieved in BCs with few positive lymph nodes, the proportion of which is largely the same for larger BCs (Engel et al. 2019; Welch et al. 2016).

### Effects of chemoprevention

Information about the growth period of BCs demonstrates that 1st/2ndBCs are prevalent over many years. Therefore, chemoprevention acts as a neo-AT for already initiated or newly during prevention initiated BCs. Since the effect of prevention already occurs within a few months (Cuzick et al. 2010) and continues even after the completion in the 5th year without risk change for more than 10 years, prevalent BCs of all sizes are most likely eradicated in 1–2 VDs of BCs, as are METs (Cuzick et al. 2015). This raises the important question of the necessary duration and the rationale of an effective prevention. Regardless of the duration, after the end of any prevention, new BCs develop because the risk of BCs continues throughout life (Fig. 2).

## Discussion

Three types of results have been considered: initiation and growth of BCs and METs with their VD times, the effects of randomized trials on adjuvant, neoadjuvant, extended and preventive therapies, and population-based data on MET-free survival along with changing MET patterns. This available evidence results in the following six statements:

First, if the start of ATs is delayed by even a few months or 1, 2 or 5 years, they will act on the then still prevalent occult METs in the same way they would if immediately starting ATs (Delozier et al. 2000; Veronesi et al. 2010). But if ATs are prolonged after 1, 2, 5 or 10 years, no comparable short-term effect on occult METs is achieved, but rather only a delayed effect after 5 and more years occurs.

If EATs cannot reduce the 30% MET from 5 to 10 years, then this should also true for the 25% METs from the 10th year. It follows that EAT does not reduce METs of the 1stBC (Fig. 3).

Second, the worse outcome after a short delay of ATs and the 5-year of ineffectiveness when AT is prolonged means, that eradicable METs are quickly destroyed and non-eradicable METs are resistant. The studies show that METs decrease in the first year, mortality delayed by MET related survival only in the third year. BCs and METs are destroyed shortly after the respective 1–2 VDs. This evidence can already be read from the studies of the 1980s with tamoxifen 1–2 years (Early Breast Cancer Trialists’ Collaborative Group 1992, 1998).

Third, EATs also act as neo-ATs on occult 2ndBCs. Three effects are to be distinguished. Most importantly, about 50% of 2ndBCs can be eradicated along with their existent and/or future METs (Blok et al. 2018; Early Breast Cancer Trialists’ Collaborative Group 1998; Goss et al. 2016). As with neo-ATs, only METs of 2ndBCs can be eradicated and thirdly, only the 2ndBC is eradicated. Resistant METs are then most commonly assigned to the 1stBC, but they are cancer of unknown, or more precisely, of already eradicated primary tumors.

**Fig. 4:**
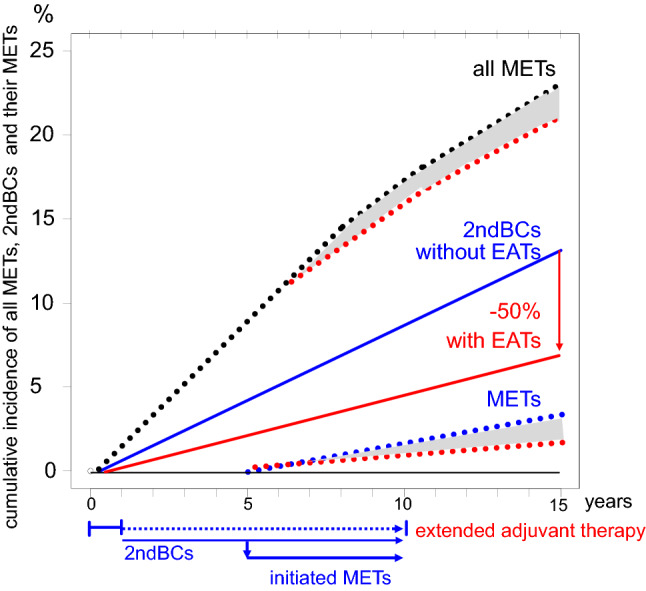
Effects of EATs by reducing METs from 2ndBCs. The cumulative incidence of METs (black dotted line) is caused by 1stBCs and 2ndBCs. If 2ndBCs (blue line) are reduced by 50% by EATs (red line), then their METs are also prevented (grey shaded area). Approximately, 4–5 years after the onset of EATs, the reduction in MET risk could become observable because of the BC’s growth variability

Fourth, the magnitude of the delayed effect of EATs seems to be the same regardless of whether ATs are extended after 1, 2, 5 or 10 years and is independent from decreasing hazard rates of METs of 1stBCs (Fig. 3). The reason is the incidence of BCs which does not vary much after the age of 55 years, at which point most BC patients are in long-term follow-up (Munich Cancer Registry; Noone et al.). Constant incidences should also apply to the 2ndBCs. Therefore, the delayed constant effect of EATs is due to the effective neo-AT on any subsequent 2ndBCs (Fig. 4).

Fifth, regarding the size of the effect, it should be noted that the risk for 2ndBCs is greater than that for 1stBCs. This increased risk is a surrogate for inherited mutations (Metcalfe et al. 2004; Michailidou et al. 2017). Just a threefold risk(Chen et al. 1999) results in a cumulative 2ndBC incidence of approximately 17% after 15 years. It fits the magnitude of these EATs effects (Fig. 4) (Chaudary et al. 1984; Gierach et al. 2016). Subgroups with classical Gail risk factors also reach such a magnitude for the 1stBC. The MET risk reduction should also be particularly large because the 2ndBCs are not yet detectable and if at all have initiated only small single METs.

Sixth, the duration of inefficiency of EATs is important to be considered. METs and subsequent BC-related death can occur 20 years after primary ATs (Fig. 3) (Pan et al. 2017). Therefore, mortality is dominated in EAT studies by the MET risk of 1stBCs with a delayed small improvement, which is postponed more than 5 years for each successive AT extension. This small EAT effect provides an insight into the tumor process: initiation of BCs up to tumor-related death can occur in HR + BCs within 10 years.

**Fig. 5:**
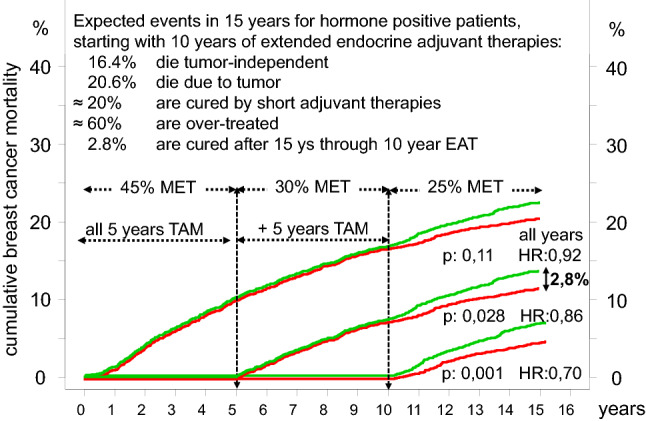
Simulations with 2 × 3500 patients with the expected mortality* and the small effect after 10 years. The survival is shown from the beginning with the common ATs, from randomization of the EAT (red, control green) after 5 years and from 10 years onward. *Mortality was simulated using the distribution in Fig. 3

EATs work because they prevent METs from contralateral 2ndBCs. The assessment of the effect is made more difficult when the cumulative incidence of METs or mortality is presented in EAT trials with a time lag up to the prolongation of AT and not from diagnosis (Davies et al. 2013). Figure 4 outlines the current incidence of METs from both 1stBC and 2ndBC, and the expected effect of an EAT extended from the first year onwards. Consequences of 10 year of EATs show a simulation with today’s survival (Fig. 5). The hazard ratio of 0.7 is formally correct from the 10th year on (Davies et al. 2013). But for shared decision making, full information must be provided about all events, in particular because the benefits of 15 years of EATs have already been pointed out (Harbeck et al. 2019). This also includes the many risks of endocrine ATs such as flush, osteoporosis, or fractures and the elaborate efforts to prevent and control side effects. Not even 50% of patients complete a 5-year therapy, indicating the burden of EATs.

EATs do not present a new mechanistic principle of action. They act like any AT, but on newly initiated, and eradicable 2ndBCs, which develop year after year in 1stBC patient cohorts. Because the historical success of AT is independent of tumor size (Engel et al. 2019; Welch et al. 2016), it is not plausible to recommend EAT depending on the risk of recurrence of the 1stBC (Burstein et al. 2019) but rather on the risk and growth rates for 2ndBCs, which should be independent of the prognosis of the 1stBC.

### Intermittent endocrine therapies

The improvement of 2.8% survival by EATs most likely comes from eradication of 2ndBCs. Immediately initiated ATs also have a chemopreventive effect on prevalent 2ndBCs and, like the METs of the 1stBCs, are quickly destroyed. Extension of ATs cannot improve the prognosis of the 1st and prevalent 2ndBCs. However, year after year, new 2ndBCs are initiated, which EATs then always act on for all future 2ndBCs. This continuous use of EAT prior to a possible future initiation of 2ndBC could be considered misuse and overtreatment. This leads us to a new treatment paradigm with intermittent treatments and the question: how short is short enough.

A change of perspective is difficult if randomized trials with extended ATs show improvements in survival. In addition, shorter treatment durations are generally cautiously discussed because hope and interest are more focused on increasing the effect with prolonged therapies (Cameron et al. 2017). In particular, early animal experiments suggested long durations for chemoprevention and ATs, which showed “…that BC development is best inhibited in the constant presence of an anti-oestrogen… and … reduction in the number and sizes of mammary tumors developing during continuous therapy” (Jordan and Allen 1980). Such a promising possibility to block the carcinogenic process can hamper. Yet in hindsight, the relation of BC and MET growth periods, the length of the therapeutic window and the duration of treatment were not as indisputably available then as they are today and were not thoroughly considered in the transfer to humans.

The available evidence suggests that after first ATs that successfully target prevalent METs of the 1stBCs as well as prevalent 2ndBCs, therapy-free intervals should follow before ATs are repeated as neo-AT against newly initiated 2ndBCs. The duration of the break has already been suggested in the ATLAS trial, because almost no METs occur in the first 5 years, a 5-year interval subdivided into 1–1.5-year treatment intervals, and 4–3.5-year treatment-free periods. ATs repeated after the break will chemopreventively eradicate 50% of new initiated BCs. The short treatment is sufficient, because the available endocrine therapies such as those that target e.g. HER2(Cameron et al. 2017) also have fast responses within few VDs times.

Such changes are already suggested. One study is testing an extended intermittent use of AI with 3 month therapy-free intervals each year after completion of 4–6 years of endocrine ATs, a tentative step towards a paradigm shift with treatment de-escalation without disadvantages for patients (Blok et al. 2018; Colleoni et al. 2018; Gnant et al. 2018). Lack of difference between 7 and 10 years of treatments also shows that shorter treatments achieve the same effects. This advancement is also suggested by prevention studies (Cuzick et al. 2015). After 5 years of prevention and an equally long therapy-free interval, a further prevention initiated from the 10th year on repeats the success. This is a convincing intermittent neo-AT of the 1stBC in high-risk patients and is equally applicable to BC patients with high risk of 2ndBCs. Only the intervals have to be adjusted.

The main question about endocrine ATs today is not “how long is long enough”(Smith et al. 2014) but “how short is short enough”, an experimental challenge that needs to be supported by further reviews and evaluation of observational data and that could promote logical evidence-based reasoning. The sequential use of aromatase inhibitors and tamoxifen should also be critically examined (Cuzick et al. 2010; Early Breast Cancer Trialists’ Collaborative Group 2015).

## Conclusion

The result of EATs is evidence-based but not the interpretation. The eradication of prevalent 2ndBCs and their future METs cause the effect of EAT and can be achieved with functionally equivalent intermittent ATs. This is logical when cause, effect and growth times of BCs and METs are considered together. Both small clusters as well as large occult foci can already be eradicated by neo-ATs at the beginning of treatment. Therefore, a reduction of treatments of 70–80% every 5 years seems possible. Shorter treatment durations would significantly reduce the many risks and improve quality of life. Considering the nearly 2 million new HR + BCs worldwide and about 14 million BC patients surviving for more than 10 years, this population burden alone obliges a critical discourse on the rationale of intermittent (neo-) ATs and a reduction of overtreatment. Improved quality of life, a modified chemoprevention for women at high risk for 1stBC and even higher survival rates after the 1stBC all appear achievable with shorter interval treatments, fewer side effects and thus better compliance.

## Abbreviations

(E)AT: (Extended) adjuvant endocrine therapy
(1st/2nd) BC: (First/second) breast cancer
MET: Metastasis
TC: Tumor cell
VD: Volume doubling
